# Disproportionate bulbar dysfunction following small corona radiata infarction in vascular parkinsonism: A case report

**DOI:** 10.1097/MD.0000000000050603

**Published:** 2026-09-11

**Authors:** Hsiao-Wen Wang

**Affiliations:** a Department of Internal Medicine, Taipei City Hospital Renai Branch, Taipei, Taiwan; b Department of Neurology, Far Eastern Memorial Hospital, New Taipei City, Taiwan.

**Keywords:** case report, cerebral small vessel disease, corona radiata infarction, dysphagia, multidisciplinary rehabilitation, vascular parkinsonism

## Abstract

**Rationale:**

Subcortical lacunar infarctions commonly occur in cerebral small vessel disease and contribute to progressive functional decline in older adults. Infarction of the corona radiata may disrupt converging corticobulbar pathways. In patients with vascular parkinsonism, diminished subcortical motor reserve may render bulbar function particularly vulnerable, resulting in neurological deficits disproportionate to lesion size.

**Patient concerns:**

A 75-year-old woman with vascular parkinsonism presented with acute dysarthria, severe dysphagia requiring nasogastric tube feeding, and marked gait instability.

**Diagnoses:**

Brain magnetic resonance imaging demonstrated a small acute infarction in the left corona radiata superimposed on extensive cerebral small vessel disease. Despite the limited infarct size, she developed profound swallowing dysfunction and impaired mobility.

**Interventions:**

Early multidisciplinary rehabilitation, including speech-language therapy, graded diet advancement, and balance-oriented gait training, was initiated and maintained throughout the 3-month follow-up period.

**Outcomes:**

At the 3-month follow-up, the patient demonstrated meaningful functional recovery, with the Barthel Index improving from 35 to 75, the Functional Ambulation Category improving from 1 to 4, and the Dysphagia Outcome and Severity Scale improving from level 2 to level 6, allowing removal of the nasogastric tube and resumption of safe oral intake.

**Lessons:**

Even small corona radiata infarctions may cause disproportionately severe bulbar dysfunction in patients with vascular parkinsonism and extensive cerebral small vessel disease. Early multidisciplinary rehabilitation was associated with meaningful functional recovery despite diminished subcortical motor reserve.

## 1. Introduction

Subcortical lacunar infarctions are a common manifestation of cerebral small vessel disease (CSVD) and contribute substantially to progressive motor impairment, gait dysfunction, cognitive decline, and loss of functional independence in older adults.^[1]^ Vascular parkinsonism (VP) is a heterogeneous syndrome characterized predominantly by lower-body parkinsonism, gait impairment, postural instability, and limited responsiveness to dopaminergic therapy. It frequently occurs in the setting of advanced CSVD and extensive white matter injury.^[2,3]^ Distinguishing VP from idiopathic Parkinson disease remains clinically challenging because of overlapping motor manifestations and the absence of universally accepted diagnostic criteria.^[2,3]^

The corona radiata represents a critical white matter hub through which corticobulbar and corticospinal fibers converge. Although infarctions in this region are often small, disruption of these strategically located motor pathways may produce neurological deficits disproportionate to lesion volume.^[4]^ Recent evidence suggests that diffuse white matter injury impairs structural connectivity, reduces neural reserve, and contributes to gait dysfunction and falls in patients with CSVD.^[4–6]^ Consequently, even a small corona radiata infarction may precipitate profound bulbar dysfunction in patients with preexisting CSVD and VP.

Because pharmacological treatment options for VP remain limited, multidisciplinary rehabilitation is a cornerstone of management for improving swallowing, mobility, and activities of daily living.^[7–9]^ However, reports describing severe bulbar dysfunction following a small unilateral corona radiata infarction in patients with VP remain scarce. Herein, we report a patient with VP who developed profound dysphagia following a small unilateral left corona radiata infarction and achieved meaningful functional recovery after early multidisciplinary rehabilitation.

## 2. Case presentation

A 75-year-old woman was admitted in August 2025 with acute-onset dysarthria, severe dysphagia, and left-sided drooling. Her medical history included hypertension, type 2 diabetes mellitus, coronary artery disease, osteoporosis, lung adenocarcinoma status post right upper lobectomy, and a left thalamic infarction 1 month earlier. She had longstanding rigidity, bradykinesia, and gait instability and had been clinically diagnosed with VP based on characteristic lower-body parkinsonian features and extensive CSVD on neuroimaging. The diagnosis was established on clinical and neuroimaging findings because dopamine transporter imaging and formal levodopa responsiveness testing were not available. Prior to this event, she was partially dependent on activities of daily living and ambulated short distances under supervision.

On admission, neurological examination revealed a National Institutes of Health Stroke Scale score of 3. Speech was markedly dysarthric, and severe oropharyngeal dysphagia necessitated nasogastric tube feeding. Motor strength was relatively preserved (Medical Research Council grade 4/5 in all extremities), with generalized rigidity but no spasticity. Sensory examination was unremarkable. Despite the limited infarct size, profound bulbar dysfunction and marked postural instability required maximal assistance for standing and ambulation. The severity of swallowing impairment appeared disproportionate to the degree of limb motor weakness.

**Figure 1.**
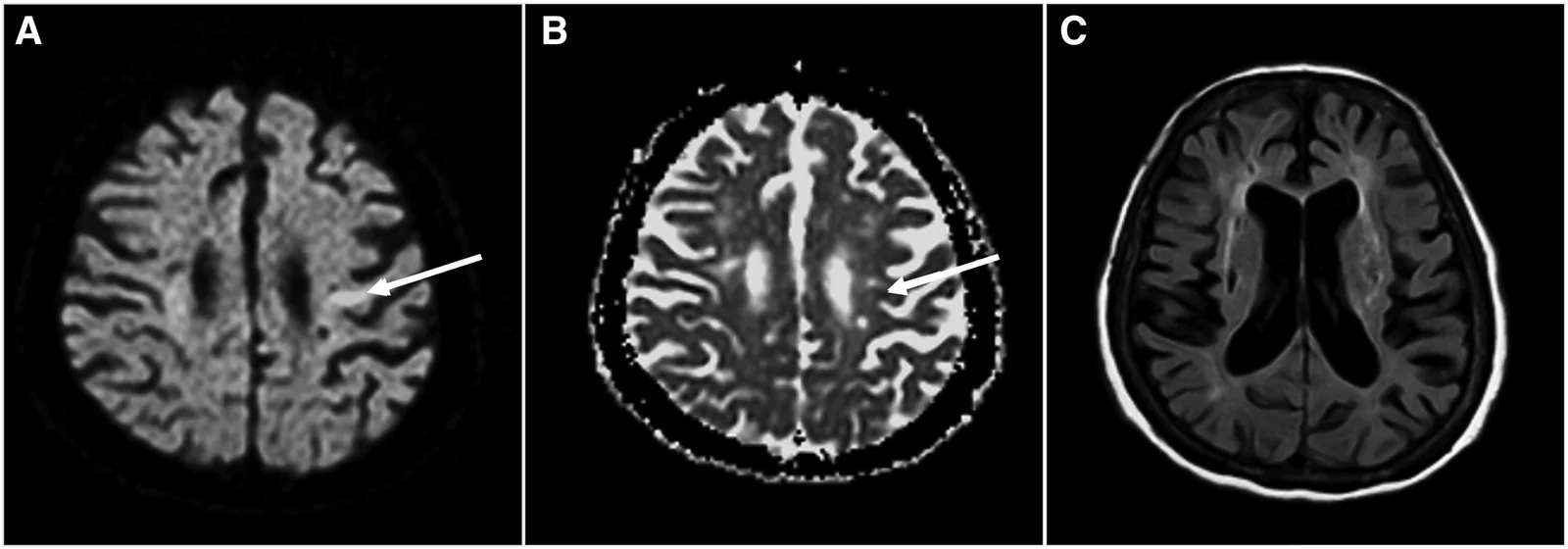
Magnetic resonance imaging of the brain demonstrating a small acute infarction in the left corona radiata in the setting of advanced cerebral small vessel disease. (A) Diffusion-weighted imaging demonstrates a small focal area of restricted diffusion in the left corona radiata (arrow), consistent with an acute lacunar infarction. (B) The corresponding apparent diffusion coefficient map demonstrates reduced signal intensity at the same location (arrow), confirming restricted diffusion. (C) Fluid-attenuated inversion recovery imaging demonstrates extensive periventricular and deep white matter hyperintensities (Fazekas grade 3) together with multiple chronic lacunar infarcts, compatible with advanced cerebral small vessel disease.

**Figure 2.**
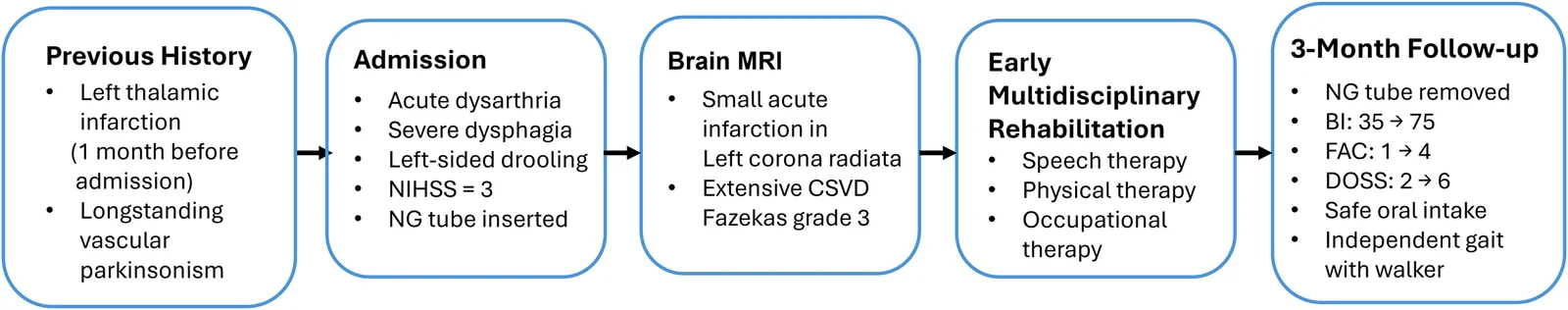
Clinical timeline illustrating the patient’s neurological presentation, neuroimaging findings, multidisciplinary rehabilitation, and functional recovery over the 3-month follow-up period. BI = Barthel Index, CSVD = cerebral small vessel disease, DOSS = Dysphagia Outcome and Severity Scale, FAC = Functional Ambulation Category, MRI = magnetic resonance imaging, NG = nasogastric, NIHSS = National Institutes of Health Stroke Scale.

Diffusion-weighted imaging demonstrated a small diffusion-restricted lesion in the left corona radiata with corresponding apparent diffusion coefficient reduction, consistent with an acute lacunar infarction (Fig. 1A and B). T2-weighted and fluid-attenuated inversion recovery sequences revealed extensive periventricular white matter hyperintensities and multiple chronic lacunar infarcts, compatible with advanced CSVD (Fig. 1C). Given the disproportionate severity of bulbar dysfunction relative to the limited infarct size, the patient was referred for comprehensive inpatient rehabilitation. The patient’s overall clinical course is summarized in Figure 2.

## 3. Rehabilitation intervention and clinical course

Early multidisciplinary rehabilitation was initiated during hospitalization and consisted of daily speech-language therapy, physical therapy, and occupational therapy delivered according to the institution’s standard inpatient stroke rehabilitation program. Therapy sessions were conducted once daily, 5 days per week, with treatment intensity and progression individualized according to serial clinical assessments and functional performance.

Speech-language therapy focused on laryngeal elevation exercises, bolus control, swallowing coordination, and graded diet advancement according to serial bedside swallowing assessments. Physical and occupational therapy emphasized trunk stabilization, transfer training, balance-oriented gait training, and functional mobility rather than isolated muscle strengthening, considering the patient’s underlying parkinsonian features and impaired postural control. Progression of the rehabilitation program was guided by improvements in swallowing function, transfer ability, balance, and gait performance.

After 3 months of multidisciplinary rehabilitation, swallowing function gradually improved, allowing progressive resumption of oral intake and eventual removal of the nasogastric tube. Mobility also improved from requiring maximal assistance for transfers and ambulation to independent ambulation with a rolling walker for short distances.

## 4. Functional outcomes

At the 3-month follow-up, functional status improved across multiple domains (Table 1). The Barthel Index increased from 35 to 75, the Functional Ambulation Category improved from grade 1 to grade 4, and the Dysphagia Outcome and Severity Scale improved from level 2 (tube-dependent) to level 6 (functional oral intake). Although mild postural instability related to the underlying VP persisted, the patient achieved independent ambulation with a rolling walker and resumed safe oral intake, reflecting meaningful functional recovery following multidisciplinary rehabilitation.

**Table 1:** Functional outcome measures at baseline (admission) and 3-month follow-up.

Measure	Baseline (admission)	3-month follow-up
Barthel Index	35	75
FAC	1	4
DOSS	2	6

FAC = Functional Ambulation Category, DOSS = Dysphagia Outcome and Severity Scale.

## 5. Discussion

This case highlights a striking clinical–radiological dissociation between infarct size and neurological severity. Although the acute lacunar infarction in the left corona radiata was limited in size and limb motor strength remained relatively preserved, the patient developed profound dysphagia and marked postural instability. The severity of bulbar dysfunction appeared disproportionate to the limited lesion size, suggesting that lesion location and preexisting network vulnerability may be more important determinants of functional impairment than infarct volume alone.

The corona radiata is a critical white matter hub through which corticobulbar and corticospinal fibers converge. Even small infarctions in this strategically located region may disrupt voluntary control of the oropharyngeal musculature, resulting in dysarthria and dysphagia despite relatively preserved limb strength.^[4,10]^

Recent evidence suggests that diffuse white matter injury may impair structural connectivity and reduce neural reserve, thereby amplifying the functional consequences of focal ischemic lesions.^[4,5]^ Characterized by rigidity, bradykinesia, gait impairment, and limited responsiveness to dopaminergic therapy, VP is associated with disruption of distributed motor networks rather than isolated focal lesions.^[2,3]^ Although these mechanisms provide a plausible explanation for the disproportionate neurological deficits observed in our patient, they remain hypothetical and are based on existing literature rather than being directly demonstrated in this case.

Poststroke dysphagia is associated with increased morbidity, aspiration pneumonia, prolonged hospitalization, and mortality.^[10]^ Current European Stroke Organisation guidelines recommend early swallowing assessment and multidisciplinary rehabilitation to optimize recovery.^[8]^ The International Dysphagia Diet Standardization Initiative framework further provides standardized terminology for dietary modification and dysphagia management.^[11]^ In our patient, early multidisciplinary rehabilitation – including speech-language therapy, graded diet advancement, trunk stabilization, and balance-oriented gait training – was associated with progressive restoration of oral intake and mobility. Improvements in the Barthel Index, Functional Ambulation Category, and Dysphagia Outcome and Severity Scale demonstrated meaningful functional recovery over the 3-month rehabilitation period. These findings are consistent with contemporary concepts of neuroplasticity, motor learning, and task-specific rehabilitation, including functional reorganization of swallowing-related motor networks during recovery from poststroke dysphagia.^[7,9,12]^

This report has several limitations. First, this is a single-patient case report and therefore cannot establish a causal relationship between early rehabilitation and functional recovery. Second, dopamine transporter imaging and formal assessment of levodopa responsiveness were not available because the diagnosis of VP had been established based on clinical features and neuroimaging findings. Third, videofluoroscopic swallowing study, fiberoptic endoscopic evaluation of swallowing, and diffusion tensor imaging or tractography were not performed, limiting objective evaluation of swallowing physiology and corticobulbar pathway involvement. Finally, the findings may not be generalizable to all patients with VP or advanced CSVD.

## 6. Conclusion

Small corona radiata infarctions may produce disproportionately severe bulbar dysfunction in patients with VP and advanced CSVD. This case highlights the potential role of impaired neural reserve and network vulnerability in neurological severity beyond infarct size alone. Early multidisciplinary rehabilitation was associated with meaningful functional recovery, although larger prospective studies are needed to clarify rehabilitation outcomes and the role of neural reserve in VP.

## Author contributions

**Conceptualization:** Hsiao-Wen Wang.

**Data curation:** Hsiao-Wen Wang.

**Validation:** Hsiao-Wen Wang.

**Visualization:** Hsiao-Wen Wang.

**Writing** – **original draft:** Hsiao-Wen Wang.

**Writing** – **review & editing:** Hsiao-Wen Wang.
